# Extended Review Concerning the Integration of Electrochemical Biosensors into Modern IoT and Wearable Devices

**DOI:** 10.3390/bios14050214

**Published:** 2024-04-25

**Authors:** Razvan Bocu

**Affiliations:** Department of Mathematics and Computer Science, Transilvania University of Brasov, 500036 Brasov, Romania; razvan.bocu@unitbv.ro

**Keywords:** biosensors, electrochemical biosensors, wearable devices, IoT devices, health monitoring, personal health data

## Abstract

Electrochemical biosensors include a recognition component and an electronic transducer, which detect the body fluids with a high degree of accuracy. More importantly, they generate timely readings of the related physiological parameters, and they are suitable for integration into portable, wearable and implantable devices that are significant relative to point-of-care diagnostics scenarios. As an example, the personal glucose meter fundamentally improves the management of diabetes in the comfort of the patients’ homes. This review paper analyzes the principles of electrochemical biosensing and the structural features of electrochemical biosensors relative to the implementation of health monitoring and disease diagnostics strategies. The analysis particularly considers the integration of the biosensors into wearable, portable, and implantable systems. The fundamental aim of this paper is to present and critically evaluate the identified significant developments in the scope of electrochemical biosensing for preventive and customized point-of-care diagnostic devices. The paper also approaches the most important engineering challenges that should be addressed in order to improve the sensing accuracy, and enable multiplexing and one-step processes, which mediate the integration of electrochemical biosensing devices into digital healthcare scenarios.

## 1. Introduction

Biosensors are generally considered relative to various real-world use case scenarios, such as clinical settings, industrial manufacturing processes, agricultural analyses, and environmental optimizations. It is worth to note that one of the earliest developments is represented by the introduction of the amperometric glucose enzyme electrode [1] in 1962. The International Union of Pure and Applied Chemistry [2] provides the conceptual description of biosensors, which is generally accepted as the most comprehensive one. Thus, “a biosensor is a self-contained, integrated, analytical device, in which a biological recognition element (biochemical receptors, including enzymes, antibodies, antigens, peptides, DNA, aptamers or living cells) is retained in direct spatial contact with a transduction element (such as electrochemical, optical, and mechanical transducers)”. Originally, the development of biosensors was triggered by the requirement to design and deploy functional point-of-care (POC) evaluation systems relative to biomolecular entities. This has permits the implementation of clinical analysis processes outside dedicated laboratories, in minimally configured public environments, such as nurseries, residential homes, or minimally equipped hospitals [3]. Several solutions regarding the development of biosensors that concern the isolation of molecules that provoke diseases are reported in the scientific literature [4]. Nevertheless, the clinical deployment (clinical translation) of biosensors is affected by the difficult integration of miniature sensors into wearable devices. It is relevant to note that the surveyed literature includes papers that propose scientifically relevant technical solutions, such as the approach that was reported in article [5] which concerns the study of electrochemical biosensors in the agrifood sector.

The fundamental aim of this paper is to present and critically evaluate the identified significant developments in the scope of electrochemical biosensing for preventive and customized POC diagnostic devices. Thus, the paper analyzes the design, integration, and deployment of amperometric, voltammetric, potentiometric, organic electrochemical transistor (OECT), photoelectrochemical, and electrochemiluminescent biosensors. The discussion is essentially linked to the diagnosis of problematic contemporary diseases, general health management processes, cell monitoring, and neuroscience. Additionally, relevant manufacturing processes are examined. Furthermore, the manipulation of biological fluids, the amplification and readouts of signals, and the related signal processing algorithms are also analyzed. The resulting visualizations which are based on the collected biological data are analytically evaluated.

The paper is structured according to the following sections. The next section discusses the significant general aspects regarding the application of electrochemical biosensors to various real-world use case scenarios, which include complex IoT and wearable device infrastructures. Furthermore, the problem of electrochemical sensing of biomarkers is analyzed. Consequently, the study approaches the scope of amperometric and voltammetric biosensors, which is followed by a discussion of potentiometric biosensors. The following sections evaluate the relevance of certain types of biosensors. The analysis is sustained by the presentation of relevant results that were obtained during the conducted experimental validation process. These are represented by organic electrochemical transistor biosensors and photoelectrochemical biosensors. This analysis is further extended by an assessment concerning the possible design and implementation of electrochemiluminescence (ECL) biosensing and bioimaging processes. Moreover, the problem of biosensor integration is discussed with an emphasis on portable electrochemical biosensing devices and the integration of biosensors into wearable devices. Additionally, relevant aspects regarding the integration of biosensors into implantable devices are also discussed. The last section concludes the paper and presents the possible development avenues.

## 2. General Remarks

**Figure 1:**
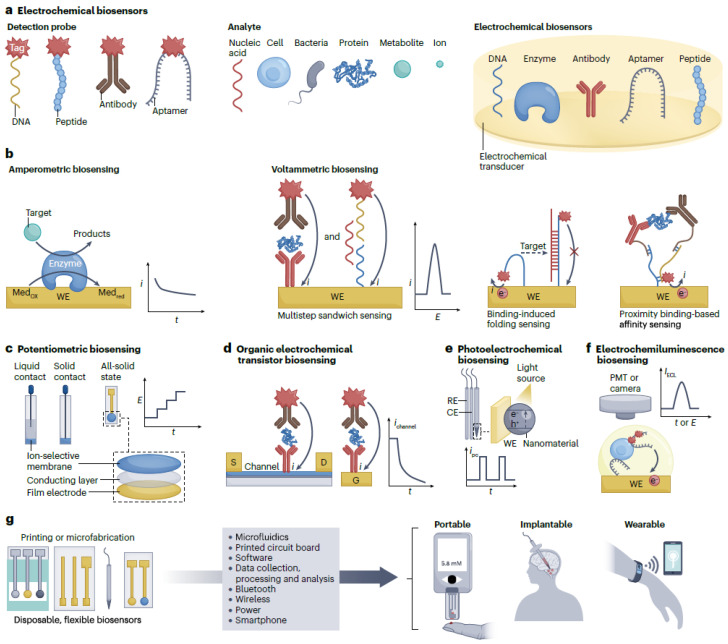
Presentation of the electrochemical biosensors.

Considering the general field of biosensors, electrochemical biosensors involve the integration of the biorecognition component into an electrochemical transducer. This can be represented by an electrode or field-effect transistor. These technical solutions are especially proper for device integration scenarios considering they are readily miniaturized and integrated on the same chip with a proper electronic acquisition module [6,7,8]. More details may be obtained by consulting the visual representation in Figure 1a, which represents a schematic description of electrochemical biosensors that relate to various biochemical receptors and detection probes. They are also easy and economical to manufacture. Moreover, electrochemical signals, for example, electrical currents and related potentials, may be gathered using plain, portable, and low-power wearable devices. It is relevant to note that the signal generated using affinity recognition of the target analyte by the biorecognition element may be enhanced through several strategies. These relate to chemical, biological, and physical approaches, which significantly enhance the sensitivity of the detection process. Therefore, electrochemical biosensors are promising concerning the design and implementation of POC devices, which may be considered relative to medical diagnosis scenarios. The World Health Organization asserts that POC biosensors should be specific, user-friendly, affordable, sensitive, rapid and robust devices, which are free and easily accessible to end users. This set of specifications has the role to enable on-site testing and diagnosis without significantly affecting the daily routines of target consumers and patients. Moreover, POC medical diagnosis is envisioned to hold a key role relative to the revolutionary diagnosis and treatment of important contemporary diseases. Thus, the electrochemical glucose meter, which is generally regarded as the most successful commercial POC biosensing device, has been widely considered worldwide in order to improve the quality of life in the case of patients with diabetes.

**Figure 2:**
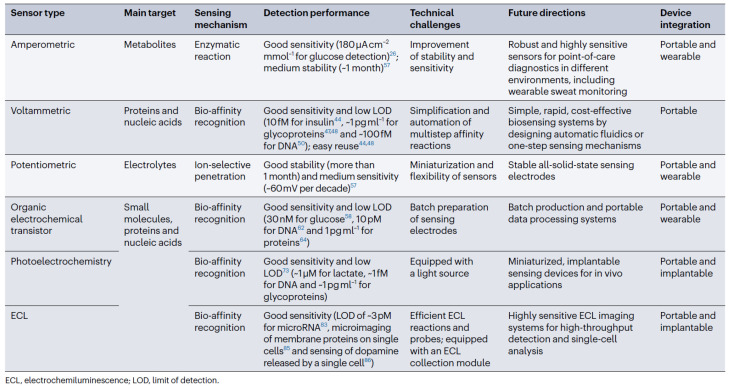
Features of electrochemical biosensors (relevant reference numbers are provided).

Nevertheless, the market of electrochemical biosensors currently offers the possibility to detect small molecules or ions. In this respect, further information is provided in Figure 2, which also specifies the related reviewed papers through their reference numbers. Contrary to this, the detection of significant biomarkers, such as bacteria, cells, proteins, and nucleic acids, essentially considers affinity recognition and needs several steps to generate a signal that can be detected. Various transduction mechanisms may favor the inclusion of fully automatic electrochemical biosensors relative to the affinity biomarkers. This may include the affinity recognition processes concerning glucose [9,10,11,12,13]. Additionally, it is also possible to consider one-step affinity-sensing processes, such as binding-induced folding sensing and proximity binding-based affinity sensing [14,15]. This is usually connected to the integration of fluid systems like pump-assisted fluidics and polydimethylsiloxane (PDMS)-based microfluidics [16,17,18]. Additionally, considering the low dose of disease markers contained in the body fluids, particularly relative to the early stages of disease (femtomolar or attomolar level), the signal amplification models are mandatory in order to increase the sensitivity of the detection. This important goal may be reached through the consideration of proper nanotechnologies and biotechnologies. As an example, proper approaches may relate to amplification strategies based on nanotags, nanocatalysis, and nanocarriers [19,20].

The design and implementation of efficient healthcare management strategies relate to the consideration of electrochemical biosensors, which are useful for both invasive and non-invasive measurement of the target molecules. The recent developments in the fields of microelectronic engineering, precision manufacture of semiconductors, and also regarding the wireless communication technologies have stimulated the inclusion of electrochemical biosensors in wearable and implantable devices [21,22,23,24] for continued detection of molecules in various biofluids like cerebrospinal fluid, interstitial fluid, sweat, saliva, urine, requires electrochemical biosensors that can be included into flexible films, textiles, glasses, teeth, and even diapers. Moreover, the hybrid systems that consider biosensors, smartphones, and other mobile devices are useful to continuously monitor dynamic physiological processes. Such approaches may also mediate the integration of intelligent data processing components into proper devices, which connect the sensors to the Internet. Such scenarios are particularly relevant in connection to real-world use cases that consider the Internet of Things and cloud computing to process and mine big medical databases.

It is significant to note that certain up-to-date technical developments that are relevant to the scope of biosensors are reported in the surveyed scientific literature. As an example, paper [25] discusses silicon transistors and metal oxide semiconductor field-effect transistors. Furthermore, paper [26] analyzes flexible electronics solutions, which are proper for the development of the biosensors technological solutions that constitute the object of this paper.

This review paper considers state-of-the-art developments that concern the electrochemical biosensors applied to prophylactic and customized POC diagnosis devices. Thus, the paper discusses the design and deployment of amperometric, voltammetric, potentiometric, organic electrochemical transistor (OECT), photoelectrochemical, and electrochemiluminescent biosensors to disease diagnosis scenarios, general health management processes, and cell dynamics evaluation. Thus, a succinct presentation of these perspectives is realized in Figure 2. Additionally, the thorough survey effort that was conducted suggested that the scientific literature also considers the broader scope of neuroscience. It is also relevant to note that this review also assesses the manipulation of fluids, the amplification of signals and their processing through efficient and algorithmically advanced models. Moreover, the visualization of the results generated by the data processing operations, which consider data collected using built-in biosensors, is also studied.

## 3. Relevant Types of Biosensors

### 3.1. Electrochemical Detection of Biomarkers

Considering the electrochemical biosensing devices, the detected signal is usually generated by the transfer of electrons or ions over a conductive transducer. This essentially generates a biorecognition process. The physical components or properties of the signals are current, potential, impedance, capacitance, conductivity, and light, which are generally determined by intensity, propagation direction, frequency or wavelength spectrum, and polarization. Thus, the impedimetric biosensors usually generate superior outcomes relative to POC diagnosis, and also concerning the integration to devices, considering that they are proper to detect biorecognition events through measurements of the non-Faradaic resistance and capacitance properties of the sensing component (electrode). This is a consequence of their ability to detect biorecognition events by measuring the resistance and capacitance of the electrode that effectively senses the physiological parameters. Nevertheless, the reported experimental evaluations suggested that their real-world usage is affected by atypical linkages of non-target compounds. This essentially determines a suboptimal level of sensitivity.

The impedimetric biosensors have been significantly enhanced during the COVID-19 pandemic. Thus, the utilization of molecular imprints to manufacture virus-imprinted impedimetric biosensors allows for a precise detection of entire virus particles [27,28]. Alternatively, the consideration of a dielectrophoresis process relates to an improvement of the detection accuracy of impedimetric immunosensors manufactured on Au micro-interdigitated electrodes [29]. Nevertheless, it is interesting to note that their functional behaviour is generally proved through the utilization of synthetic physiological samples, which replace the real-world clinical samples.

The accuracy and reliability of electrochemical biosensors may be enhanced through the consideration of biosensor arrays. These sensibly improve the detection process relative to various environmental conditions. The manufacturing of these arrays may benefit from all-solid-state electrodes, which have the advantage of efficient integration into printed circuit boards. The biosensor arrays generally produce convoluted signals, which may be decomposed (deconvoluted) through specific algorithmic models, such as Fourier and wavelet transformations.

The following subsections describe each particular category of biosensors, and relevant technical examples and data are provided. The experimental numerical values are obtained and calibrated using the infrastructure of the Research Institute, Transilvania University of Brasov, Romania, and also data provided by relevant industrial partners.

### 3.2. Voltammetric and Amperometric Biosensors

Voltammetric and amperometric biosensing devices function using a system with three electrodes. This includes a biosensing device in the form of a working electrode (WE), which detects the target entity, a counter electrode that generates the current, and a reference electrode that induces a stable potential. The signals of the current are produced by electrochemical reactions on the WE, which are generated by a properly applied potential. The voltammetric detection is different from amperometric measurements considering the applied potential, which is constant for amperometric biosensors, and variable for voltammetric biosensing devices. More precisely, the measurements that relate to three electrodes are based on the usage of a potentiostat.

Amperometric biosensing devices are mostly used to detect metabolites, such as glucose, lactate, and uric acid. Thus, an enzyme that is specific to the target, such as glucose oxidase (GOx), lactate oxidase, or urate oxidase (uricase), is applied on the WE as a catalyst to the oxidation of the target [6]. As an example, glucose meters are usually manufactured with amperometric biosensing components that employ GOx to catalyze the oxidation of glucose. Thus, more details are visually presented in Figure 1b relating to an amperometric biosensing of metabolite targets based on an enzyme electrode. This includes the current–time (*i–t*) curve, and the *i* signal to obtain the quantitative measure. This figure also suggests that voltammetric biosensing of proteins or nucleic acids may be conducted using an antibody-modified or nucleic acid-modified electrode through a multistep sandwich detection process. As an implementation alternative, amperometric glucose sensors may consider the enzymatic oxidation of glucose with natural oxygen. This both detects and generates hydrogen peroxide using a mediating substance, such as Paris blue [30]. The manufacturing process of amperometric biosensing devices is relatively simple. Additionally, they are usually featured by a high level of sensitivity, and they efficiently select the intended target. This ensures their suitability as basic elements of real-world wearable applications. It is relevant to note that nanomaterials, such as metallic nanoparticles, carbon nanotubes, and graphene, may be applied to these biosensors to mediate the electron transfers, which helps to increase the sensitivity and decrease the limit of detection [31]. As an example, such a biosensing device may be suitable to construct a smart contact lens, which measures the concentration of glucose from tears [32].

In addition, nanomaterials that act as artificial nanoenzymes may be constructed as part of amperometric biosensing components in order to prevent the degradation that is characteristic of natural enzymes. The reviewed literature reports the integration of such a biosensor with portable stations built using smartphones, which are considered for measuring the levels of glucose relative to commercial devices [33].

Metabolites may be determined through an enzymatic detection process. In contrast, disease biomarkers like proteins and nucleic acids are determined by affinity recognition. This method cannot directly produce electron transfers on the biosensor’s surface. Therefore, a supplementary electroactive label is required relative to the target sensing entity, such as enzymes like horseradish peroxidase and alkaline phosphatase, nanomaterials like nanoparticles, nanotubes and quantum dots. Additionally, in this context, it is also worth to mention electroactive molecules like ferrocene and methylene blue. The respective labels are usually determined through cyclic voltammetry, differential pulse voltammetry, square wave voltammetry, and anodic stripping voltammetry. Consequently, the affinity sensors are, in general, voltammetric biosensing devices. The manufacturing of such sensors may relate to the immobilization of capture biomolecules, such as antibodies and antigens, on the WE [34,35,36]. This approach mediates the detection of nucleic acids or proteins through a sandwich assay format. Thus, the process is visually presented in Figure 1b.

The science of microfluidics studies the behaviour of fluids through micro-channels and the technology of manufacturing microminiaturized devices that include chambers and tunnels through which fluids flow or are confined [37]. Consequently, liquid processing models that consider multiple stages may be built into a single chip, which performs the full analysis process, from the initial sample analysis to the generation of the required result [38,39]. While these specialized products allow for the assessment of disease biomarkers at home, they are very expensive. As an alternative, economically effective and sensitive electrochemical biosensors may be designed through a combination of amperometric or voltammetric biosensing components with paper-based microfluidics components, which feature self-pumping capabilities. These functional solutions may include multiplex sensing electrodes, which relate to differential pulse voltammetry, or square wave voltammetry detection processes that relate to proteins and nucleic acids biomarkers [16,18]. As an example, the reviewed literature suggests that an origami paper-based aptamer and antibody biosensing chip mediates the simultaneous detection of C-reactive protein (CRP) and pre-albumin down to the picogram per millilitre level [40,41,42,43]. Additionally, an interesting approach concerning the detection of breast cancer biomarker was reported in article [44].

The combination of biosensing devices with automatic fluid devices facilitates the detection process. Nevertheless, complete device integration stays problematic considering the necessity to also deploy pumps and reservoirs. Thus, one-step affinity biosensing devices offer a simpler solution. As an example, a binding-induced folding electrochemical biosensing device may be manufactured through specific changes applied to a redox-tagged DNA probe over the already presented working electrode (WE) [14]. Consequently, the detection of the target element relates to the binding-induced change of DNA probe rigidity. The process is visually presented in Figure 1b. This class of binding-induced folding electrochemical biosensing devices may produce faster and more accurate answers using aptamer receptors. Nevertheless, they exhibit a general low sensitivity. The related signals may be enhanced using approaches that are based on DNA hybridization [45,46]. For example, there is an electrochemical DNA biosensor considers target-induced CRISPR–Cas12a cleaving of interfacial single-stranded DNA with methylene blue. This is based on the assumption that the related signal can detect human papillomavirus 16 (HPV16) and parvovirus B19 (PB19), going all the way down to the picomolar level [47]. It is relevant to note that the sensitivity of the DNA biosensor may be improved through the consideration of a hairpin DNA probe, as it was demonstrated in paper [48].

A voltammetry biosensing device which considers the wash-free and separation-free square wave technique is used to implement the direct detection of insulin [49]. The inclusion of uracils (one of the four nucleobases in the nucleic acid RNA) in the DNA sequence determines a reusable biosensor, which also provides fast detection times [50]. The detection capabilities of proximity binding-based affinity electrochemical biosensors may also be enhanced through the consideration of DNA amplification strategies. Thus, interesting contributions in this respect were described in papers [51,52,53,54,55,56].

Thus, we determined that a concentration of metabolites in non-blood fluids that is lower than the concentration of metabolites in blood (e.g., a concentration of sweat glucose that is between 10 and 200 μM or glucose in tears that is between 0 and 2 mM, which are, respectively, 100-fold and 10-fold lower than that of blood glucose (1–20 mM)) implies that nanomaterials like metallic nanoparticles, carbon nanotubes and graphene, may be considered as part of the biosensing interface. This would facilitate the transfer of electrons in order to increase the level of sensitivity, which would imply the existence of lower detection limits. As an example, Au-Pt bimetallic nanocatalysts coupled with nanoporous hydrogels activate GOx immobilization and glucose detection with a sensitivity of 178 μA${\mathrm{cm}}^{-2}$${\mathrm{mmol}}^{-1}$ and a detection threshold of 0.01 mg dL^−1^ (0.56 μM). Considering the technical experiments that were conducted, it is relevant to note that this level of technical performance opens ways for interesting real-world use case scenarios. Thus, these biosensors may be considered to construct smart contact lenses used to measure the level of glucose in tears.

It is also relevant to note that artificial nanoenzymes may be constructed relative to amperometric biosensors, which would prevent unwanted changes in the structure of natural enzymes. The experiments that were conducted demonstrated that the consideration of a laser-induced graphene array coupled with Cu_2_O and Au nanoparticles mediated the implementation of an electrochemical, flexible, miniature, and non-enzymatic biosensor. This generated precise and stable detection signals over 52 application–removal cycles. Let us recall that this result was obtained in the experimental setting that was set up while the surveyed literature reports certain lower numerical values. Thus, paper [32] reported a stable utilization of such biosensors over 25 application–removal cycles. The reported inferior performance may be determined by particular implementation glitches or by certain suboptimal materials that were used to construct the biosensors.

### 3.3. Potentiometric Biosensors

Potentiometric biosensing devices generally consider a two-electrode system, which relates to a sensing electrode, and a reference electrode. These allow direct detection of targets using the value of the potential signal concerning the change in surface charge on the target recognition of the sensing electrode. Usually, these consider ion-selective electrodes made of ion-selective membranes and a liquid contact structure that determines the potentiometric sensing electrodes. More details are provided in Figure 1c, which represents ion-selective electrodes featured by three different structures. The process includes the recording of potential (E) for the determination of quantitative values. Thus, this functional model is able to detect enzymes, nucleic acids, and proteins through the integration of the respective biological compound over the ion-selective electrode. This acts as a catalyst of the reaction that generates the ions through the combination of the target event with an ion-based reaction [57,58,59,60].

It is important to note that solid-contact ion-selective electrodes are not based on internal solutions. They are reliable and exhibit morphological diversity, while simple manufacturing processes offer ample possibilities for miniaturization. Solid-contact ion-selective electrodes mediate the analysis of proteins and nucleic acids relative to the detection of ions generated by probes tagged with nanoparticles. As an example, a miniature solid-contact Ag ion-selective electrode can be used for the detection of DNA targets down to the femtomolar level. Such analyses consider microlitre-level samples [61]. Additionally, ion-selective electrodes that are all-solid-state can be manufactured with conductive polymers or proper nanomaterials, which realize a reliable contact under the ion-selective and reference membranes. More information is visually presented in Figure 1c.

It is interesting to note that a paper potentiometric biosensor, which is based on an all-solid-state butyrylcholine-sensitive ion-selective electrode, and a 3D origami paper-based fluid system may be used to detect butyrylcholinesterase activities and also organophosphate pesticides. This useful model is further enhanced by the inclusion of a miniature USB electrochemical analysis element. This essentially creates adequate conditions for the implementation of a handheld potentiometric device [62].

During the conducted experiments, we considered two commercial portable devices to detect electrolytes and blood gases in POC settings. The respective devices are i-STAT from Abbott and BGA-102 from Wondfo Biotech. Additionally, all solid-state electrodes may be built into wearable devices for the purpose of detection of ions in biofluids. Thus, a smart wristband featured with Na^+^ and K^+^ ion-selective electrodes was considered during the experiments to evaluate Na^+^ and K^+^ in sweat through an in-place (in situ) approach.

### 3.4. Organic Electrochemical Transistor Biosensors

Organic electrochemical transistor biosensors (usually abbreviated as OECT) are organic thin-film transistors, which include gate (G), drain (D), and source (S) electrodes. There is an organic semiconductor film between the D and S electrodes. The operating principle implies that a potential drop change or a capacitance change modify the detected channel current. As a consequence, OECT biosensing elements may be manufactured through the immobilization of the detection component relative to the G electrode, or directly over the channel’s surface, as it is suggested in Figure 1d, which displays two types of organic electrochemical transistor sensors prepared by immobilizing the recognition element on the channel surface or on the gate electrode (G). Thus, the particular feedback of the OECT biosensing element relative to the target determines the potential of the interface. This essentially generates a quantitative measurement of the channel’s current feedback.

Thus, OECT biosensing elements provide a high level of sensitivity, low manufacturing and implementation costs, and also low working voltages of less than 1 V. This mediates the detection of electroactive molecules, such as dopamine, glucose, and epinephrine [63,64,65], and also electroinactive molecules, such as cortisol [66], DNA [67], proteins [68,69], bacteria [70], cells [71], and also glycans [72,73,74,75]. Additionally, the reviewed scientific literature reports contributions that describe the electrostatic interactions or affinity binding between targets and the respective sensing interfaces [76].

The OECT biosensing components may be readily miniaturized and consequently integrated into IoT or general wearable devices. This is particularly possible considering that their detection performance is not adversely influenced as their size reduces relative to a constant channel width per length ratio. As an example, a lab-on-a-chip (LOC) device that includes an OECT biosensor may detect DNA molecules with a limit of 10 picometres. Thus, the microfluidic device is stored over a flexible substrate that contained a thiolated DNA probe fixed on the Au gate electrode. Additionally, OECT microarrays may also be manufactured by solution processes that pertain to high-throughput sensing. As an example, the biosensors may be included into a diaper to assess the glucose levels found in urine, while the collected signal levels are displayed and stored on a mobile phone. The data transfer is conducted using the Bluetooth interface [77].

### 3.5. Photoelectrochemical Biosensors

The scientific field of photoelectrochemistry assesses the effects of light on photoelectrodes and photosensitive materials, as well as the processes of sun light conversion to electricity. Photoelectrochemical biosensors blend photoelectrochemistry with sensor-based bioanalysis. Thus, light serves as the source of excitation, and the generated electrical current constitutes the produced data readout. Photoelectrochemical biosensors generally relate to three electrodes and a source of light, as it is also suggested in Figure 1e, which relates to a photoelectrochemistry biosensing process based on a three-electrode system and a light source. This includes the storage of the photoelectrode photocurrent’s values. The detection is possible considering the modification of the photocurrent on the determination of the target at the surface of the biosensing component. This essentially creates a charge or energy transfer that is determined by the photoelectrochemical reaction between a donor electron, an acceptor, and a photoactive material placed on the surface of the electrode [78].

Photoelectrochemical biosensors provide a functional hybrid between the advantages of optical and electrochemical assays. These may allow their efficient usage for the detection of disease molecules, such as glutathione, lactate, DNA, microRNA (miRNA), protein tumour markers, and cells [78]. The light stimuli can be generated contactlessly, which suggests photoelectrochemical biosensors as reliable technical solutions for in vivo sensing. Moreover, the distinction between the excitation source of light, the detection electrical signal, and their different energy patterns may produce a scenario featured by low background noise and high sensitivity. This suggests that photoelectrochemical biosensors are proper for in vivo or single-cell analysis [79,80]. The contribution that was reported in paper [81] relates to an interesting real-world use case, which evaluates cerebral ischemia in the brain of the target living rats. The considered fluorescence resonance energy transfer (FRET) model relates to a photoelectrochemical microbiosensing system that can selectively monitor SO_2_. Moreover, the FRET mechanism was implemented using upconversion nanoparticles (UCNPs) with the role of energy source, while an organic dye played the role of an energy receptor. Therefore, the surface that acts as a biosensor was built through the simultaneous immobilization of the UCNP and FRET pair, and also using Cadmium telluride (CdTe) crystalline compounds placed on the microelectrode. Thus, relative to a rat brain affected by cerebral ischaemia-reperfusion and febrile seizure, the SO_2_ blocked the FRET process and reactivated the generation of UCNP. This modulated the photocurrent that passes through the photoactive material, which effectively implements the detection of SO_2_.

### 3.6. Biosensing and Bioimaging Based on Electrochemiluminescence

Electrochemiluminescence (ECL) is an energy relaxation process that is triggered electrochemically, in which a luminophore goes through a transfer of electrons. The process generates excited states that produce light. Thus, ECL biosensors are proper for the quantitative detection of specific molecules using ECL emission signals. Comparable to amperometric and voltammetric biosensors, ECL components consider three electrodes. The working electrode is customized so that the detection component acts as the biosensing electrode, as it is suggested in Figure 1f, which displays the ECL biosensing of cells using an aptamer-modified electrode. The approach is connected to a sandwich-sensing format. Considering the hybrid detection model that relates to electrochemistry and spectroscopy, these biosensors work in the absence of light. They generate very low levels of background noise, and they are highly sensitive. These features recommend electrochemiluminescent biosensors as efficient analysis instruments for the determination of various disease molecules, such as DNA, miRNA, proteins, and tumour cells [82,83,84,85,86].

Usually, ECL biosensing setups may be complemented by a charge-coupled camera device, and also a conventional microscope that conducts the actual bioimaging. This system allows for the concurrent detection of several biomarkers using spatial or potential resolutions. Thus, there are related immunosensing arrays that concurrently detect three antigens through the individual detection of the microbeads located in a microwell array [87]. A logically related contribution was described in paper [88].

It is relevant to note that ECL biosensors are suitable for the implementation of cell analysis setups, as they generate both morphological and quantitative information [89]. Therefore, these sensors are routinely applied to various targets that include small molecules released from cells and membrane proteins that adhere the surface of the cell [90]. The quantitative analysis of the detection process considers the biosensing interface. This may consist of a Pdot-modified-indium tin oxide (ITO) glass electrode sheet, which is merged with a single-cell capture microfluid chip [91]. This enables the implementation of a high-throughput quantitative analysis model relative to the dopamine produced by a single cell.

The experiments that were conducted considered a commercial ECL biosensing system, which is Elecsys 1010/2010/E170 produced by Roche Diagnostics. This is generally regarded as one of the most accurate detection systems that are used in clinical environments relative to glycoprotein tumour markers. Nevertheless, we determined that this accurate device is large, which may pose practical problems in certain real-world use case scenarios. Therefore, we experimentally implemented a portable ECL device which is based on a screen-printed carbon electrode-based ECL biosensing component, paper microfluidics, and a mobile phone camera. This miniature system can detect 2-(dibutylamino)-ethanol and NADH. Complementary, the experimental process also considered the design and implementation of a portable ECL biosensing device, which is compatible with the detection of the mammalian microRNA miRNA-21.

It is also relevant to note that electrochemiluminescent polymer dots (Pdots), luminol-doped Pdots, and diethylamine-coupled Pdots can be considered for the high-throughput detection of miRNAs. Thus, luminol-doped Pdots exhibit electrical voltages of +0.6 V, while diethylamine-coupled Pdots generate electrical voltages of +1.0 V.

## 4. Integration into Wearable Devices

Electrochemical biosensing components may be built into portable, wearable, or implantable devices, as suggested by Figure 1g, which presents the integration strategies of electrochemical biosensors into portable, wearable, and implantable devices. Relative to this figure, the following components may be observed: CE (counter electrode), D (drain electrode), $Med_{ox}$ (oxidized form of mediator), $Med_{red}$ (reduced form of mediator), RE (reference electrode), S (source electrode), and WE (working electrode). Thus, amperometric biosensors are mostly used for the analysis of metabolites. Considering the particular enzymatic reactions, they generally produce a sufficient level of selectivity. Additionally, the enzymatic catalytic signal may be improved through the usage of nanomaterials, which generates a superior sensitivity. These enzymatic biosensors are configured in batches, and the process is easily reproducible. Nevertheless, the enzymatic dynamics may be affected by certain features of the environment. Therefore, it is necessary to design reliable sensing electrodes that are proper for various environmental conditions.

Potentiometric biosensors may be considered as components of wearable sweat monitoring systems, which specifically analyze the electrolytes. Relative to the ion-selective membranes, the potentiometric biosensors exhibit the necessary selectivity, reproducibility, and stability. Their main disadvantage is represented by the low sensitivity. By contrast, a flexible all-solid-state wearable ion-selective electrode may sustain a continuous sweat monitoring process. It is also relevant to note that voltammetric, photoelectrochemical, OECT, and electrochemiluminescent biosensing components are proper for the determination of proteins and nucleic acids, considering their physical capacity to select, and also their high sensitivity. Nevertheless, they are more difficult to manufacture than the enzyme electrodes.

### 4.1. Portable Electrochemical Biosensing Devices

These have been initially proposed to measure the levels of glucose relative to patients with diabetes [92]. The personal glucose meter represents a portable electrochemical biosensor that offers a quick quantitative analysis of the glucose levels in blood. Technically, this is generally an amperometric biosensor that relates to a redox enzyme. It features a disposable test strip and a handheld electrochemical reader. The disposable test elements may be manufactured using economically effective materials like plastics and conductive pastes [93]. The glucose meter is the typical example of a biosensors that has been continuously improved in order to improve its reliability, accuracy, and user friendliness [94,95,96].

**Figure 3:**
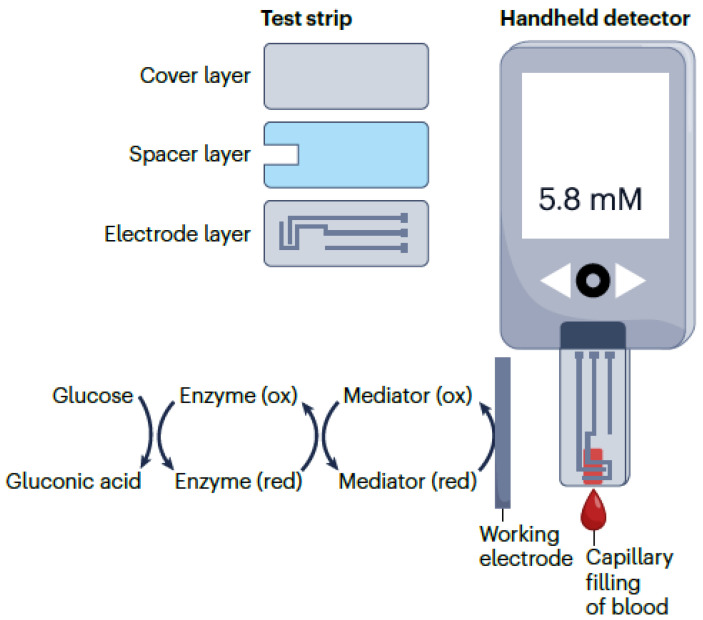
Functional architecture of portable glucose meter.

It is also relevant to note that the personal glucose meter may also be considered for the detection of metal ions, drugs, enzymes, proteins, organic metabolites, DNA, and influenza viruses. The detection mechanism analyzes the reference detection events relative to the usage of glucose, considering both its generation and consumption [97,98,99,100,101,102]. Relevant real-world use cases imply that the glucose meter can detect cocaine, uranium, and even adenosine in related blood samples [97]. Additional relevant contributions were reported in articles [103,104,105,106,107,108,109,110]. Thus, the functional logic of the personal glucose meter is presented in Figure 3, which suggests that the portable blood glucose meter is composed of a handheld electrochemical detection sensor and disposable test strips. These contain a bottom electrode layer, an adhesive spacer layer, and a hydrophilic cover layer. The blood sample enters the reaction cell through capillary force.

Miniature electrochemical biosensors may also be linked to smartphones. Mobile devices may be used as data processing and storage devices. They may even provide power to other subsystems and display the results of data processing operations [111]. Additionally, the results may also be uploaded to various e-health distributed infrastructures using the proper data privacy mechanisms [112,113]. Thus, relevant real-world field deployments consider the connection of a portable electrochemical biosensors to a mobile phone using the Bluetooth interface or another similar wireless protocol [111,114].

### 4.2. Relevant Wearable Biosensors Integration Scenarios

Wearable biosensors may be linked to smartwatches, bracelets, glasses, and other similar wearable devices to monitor the health parameters, such as the heart rate and the related electrocardiogram variables [115,116,117,118]. It is also relevant to note that it is algorithmically possible to convert the concentration of glucose relative to other types of fluids like sweat and tears [119,120,121]. Relative to portable electrochemical biosensors, wearable electrochemical biosensors are not at the same level of technological readiness.

Thus, wearable biosensors are mostly used to detect levels of glucose in the related blood samples and also relative to saliva, sweat, and tears [122,123,124]. The non-invasive nature of wearable glucose meters brings clear advantages both considering the continuous monitoring of the glucose levels and also the comfortable application for the end users [125,126,127,128]. The technique of sampling is relevant concerning biosensing processes that are based on wearable devices. The concentration of target molecules in the body fluid samples is influenced by several phenomena such as evaporation, secretion rate, substances that interfere with the detection process, reabsorption, and metabolism of the secretion glands [115,119,129]. Wearable devices that are designed to process microfluids may be used to collect biological samples. Thus, sweat may be processed to the biosensing component of a relevant device, which would reduce the proportion of sweat that evaporates or is reabsorbed. This strategy supports the implementation of real-time monitoring systems. Additionally, this solution may also allow for the collection of small biological samples, which implies that the monitoring process can be accomplished at rest [130]. This architectural solution is proper for monitoring systems that are compatible with the end users’ daily activities, including physical exercise. These may also allow for the assessment of relevant biomarkers’ time-induced variation [131]. The reviewed literature also describes epidermal microfluidic devices featured with thermosensitive hydrogel valves, which implement the active control of sweat [132]. This is accomplished by the on-demand transportation of sweat molecules to the implied biosensor, which circumvents the problematic variable flow rate and is consequently compatible with the implementation of scheduled sweat analysis scenarios.

Nevertheless, empirical studies report that certain problematic aspects should be addressed. Thus, it is difficult to implement these devices as they need to undergo multiple processing stages regarding incubation, amplification, and washing. This sensibly limits their usefulness to monitor protein and nucleic acid biomarkers that are found in sweat. Consequently, it is necessary to develop more efficient biofluids control units, which would sustain automatic multistep bioassays processes.

It is relevant to note that a power source is mandatory for the continuous bioanalysis based on electrochemical wearable devices. The surveyed literature describes wearable devices that are self-powered and produce the necessary energy out of human motion [133,134,135]. This is accomplished through a piezoelectric nanogenerator [136,137] or a triboelectric nanogenerator [138,139,140] that converts mechanical energy into electrical energy. Further relevant technical solutions were reported in papers [141,142].

As an alternative, biofuel cells can produce the required power for the wearable biosensors based on the redox substances found in biological fluids through bioelectrocatalytic reactions [134,143,144]. Thus, a self-sufficient contact lens can measure the glucose levels found in tears [145]. In a similar way, a self-sufficient wireless sensing system that uses glucose and lactate biofuel cells may detect the glucose levels in sweat and also the lactate levels [146]. There are cases when a power source is not enough to power the device. Consequently, a microgrid system that includes biofuel cells, supercapacitors, and triboelectric generators may generate the required power output [147].

There are wearable electrochemical biosensors that are designed for long-term usage scenarios. These may consider flexible electrode materials, such as certain metals and conductive polymers, which are resistant to mechanical deformations and are able to self-heal [148]. Additionally, it is possible to design flexible printed circuit boards. These include fully featured microcontrollers, communication modules, and other necessary electronic components [149]. Certain wireless data transmission protocols, for example, Bluetooth [150,151] and near-field communication (NFC) [152,153], feature low power requirements over sufficiently long distances. This allows for the biosensing devices to transmit collected data to remote appliances, such as mobile phones, which are able to display, analyze, and store the data. If necessary, these may also send the data further to relevant distributed or cloud-based infrastructures [112].

Nevertheless, the real-world behavior of wearable biosensors is affected by variable levels of connectivity and also by the electrical impedance generated by human activities, which may provoke inaccurate detection instances. The reviewed literature describes certain algorithmic solutions and calibration techniques which have the role to recover these errors [121]. Certain relatively advanced algorithmic models, such as wavelet-transform projection, may be considered to separate motions from the actual electrochemical detection process [154]. The relative variation of the electrochemical signal may be considered to the detriment of the absolute value of the signal to attenuate the measurements’ error levels [122].

### 4.3. Implantable Devices

Implantable devices designate a category of wearable devices that include various biosensors in real-world scenarios. Thus, implantable electrochemical biosensing components blend the high accuracy of invasive finger-prick assessments with the long-term monitoring model of non-invasive evaluations based on wearable devices [124,155]. Implantable biosensing devices are usually considered for monitoring the levels of glucose [125,126] and also for the detection of certain biomarkers, such as neurochemicals that are found in the brain [127,156].

Relative to electrochemical biosensors, the detection takes place on the surface of the electrodes. Therefore, these sensors may be integrated into an implantable capsule along with the necessary electronic circuitry [157,158]. Implantable biosensing devices, such as intravascular or subcutaneous, may offer real-time or near-real-time updates regarding the levels of glucose. This may further support the proper adjustments of clinical treatment regimens [128,129,159]. Additionally, spatiotemporal electrochemical detection of neurochemicals, such as dopamine and acetylcholine, may effectively indicate the pattern of brain activity and help to discover any potential abnormalities [130,160,161].

The mechanical and general physical incompatibilities between implantable biosensors and the target tissuesmare also studied in the surveyed literature. These may lead to the failure of the implanted devices or even to problematic or life-threatening inflammatory reactions. Consequently, the implanted components (electrodes) should be sufficiently soft and flexible in order to ensure the maximum possible physical compatibility with the target tissues [162].

Implantable biosensing devices usually stay in the body over a long time period. This requires proper reliable power supply [136] which is featured in a high-density energy storage model [137,138]. Batteries fulfill the energy storage density constraints, but need to be replaced periodically. This poses easily discernible medical risks, such as the possible infection of target tissues [163]. Therefore, as it has already been mentioned, it is possible to manufacture self-sufficient implantable biosensors which generate the necessary power using piezoelectric materials, triboelectric materials, or fuel cells [163,164]. It is also interesting to note that electrical power may be generated wirelessly through the consideration of technical solutions that pertain to the scope of near-field communication [139]. In such a scenario, the power transmission channel may also act as the data transmission link [140].

### 4.4. Considerations Regarding Resource-Constrained Wearable Devices

The wearable biosensing devices that are designed for point-of-care (POC) evaluation scenarios should be easy and economical to manufacture, portable, friendly to the end users, and provide timely results. Naturally, the aspects that pertain to data storage and to the system’s long-term stability should also be analyzed. Thus, electrochemical biosensing devices may be manufactured in the form of disposable test strips. The data readouts can be gathered and consulted using a handheld reader. The necessary test strips, such as glucose-sensitive test strips, usually feature a shelf life of a few months in ideal room temperature and dry conditions, which mediate the transportation and storage in the absence of special cool chains.

This plain architectural model guarantees compatibility with large-scale and economically effective industrial manufacturing scenarios. Wearable devices that use test strips offer sufficiently accurate and fast analysis results, even in the absence of qualified personnel. Moreover, the integration of electrochemical biosensing devices with smartphones, watches, and wristbands is proper for conducting home analyses of biophysiological molecules. Therefore, they can be perceived as valuable tools for health monitoring and disease diagnosis.

### 4.5. Remarks Concerning Translational Research Processes

Translational research processes require the specification of precise diagnostic criteria which are used to evaluate the results of the tests conducted relative to various types of samples. As an example, the testing procedure concerning glucose tests are thoroughly specified relative to blood samples. Nevertheless, the technical specification of the testing procedure is not sufficiently specific regarding other biological fluids, such as sweat, saliva, and tears. Moreover, relative to blood samples, these types of biological fluids may be influenced by sampling location. For example, saliva may be collected from different portions of the mouth, and the collected sweat may be produced by different glands. The dynamics of the environment may also affect the uniformity of the samples. As an example, the biological samples may be collected before or after physical exercise, or relative to the moment when water was drunk. Consequently, the reviewed scientific literature suggests that it is still difficult to design a fully comparable evaluation system for target biological samples.

Additionally, the criteria that define such tests are not fully and clearly specified. Therefore, the translational research processes that involve blood samples may substantially differ from the translational research processes that relate to other types of biological fluids. Therefore, it is imperative to infer that it is necessary to standardize the sample processes of biological fluids. This may be accomplished, for example, by the inclusion in the process of additional biosensing and detection units to monitor the variation of pH values, temperatures, and flow rates of the target biological fluids. Consequently, the enhanced analysis process may benefit from a consistent phase of calibration.

For example, the initial commercial versions of blood glucose meters concerned the measurements conducted in hospital settings, which clearly suggested the necessary technical specifications for the biosensing devices. In contrast, contemporary wearable biosensing devices designed for other types of biological fluids may not benefit from a consistent empirical process conducted in hospitals, as they are mostly evaluated and validated as consumer devices. Therefore, further conceptual and experimental work should be conducted in order to fully validate the most recent wearable biosensing devices in real-world medical scenarios.

## 5. Conclusions

Electrochemical biosensors have been continuously and intensely enhanced during the past forty years, although the first functional biosensors for oxygen detection was proposed in 1956 by Leland C. Clark. The sustained research and practical efforts generated increasingly complex technical solutions, which also tend to fail more often than the past biosensing devices. The sensitivity of the detection process and the selectivity essentially relate to the recognition reaction that occurs on the delicate electrolyte–electrode interface. The operation of this component is influenced by several factors, like the friction between electrodes and biological tissues and the variation of pH values. The flow rate of the biological fluids and the related temperatures are especially important relative to wearable devices. Consequently, the robustness and reliability of the electrochemical biosensors should be improved, which would allow the design and implementation of long-term health monitoring systems. Thus, the reviewed literature suggests that the principle of enzymatic chemistry may be replaced by solutions related to nanomaterial-based catalytic sensing chemistry, which is less prone to be affected by environmental conditions like temperature, pH, and ionic strength.

Moreover, electrochemical biosensors may be made more accurate and reliable through the implementation of biosensor arrays. These sensibly improve the detection process in various environmental conditions. The manufacturing of these arrays may consider all-solid-state electrodes which have the advantage of fast integration into printed circuit boards. The biosensor arrays generally produce convoluted signals, which may be decomposed (deconvoluted) through specific algorithmic models such as Fourier and wavelet transformations. This approach is useful to implement efficient concurrent detections of biological parameters. The improvement of the accuracy may be realized using approaches that are generally used relative to electromyograms, electrocardiograms, and magnetic resonance imaging. This may involve the compressed detection of signals through the consideration of a sampling rate that is lower than twice the value of the highest recorded frequency of the detected signal.

This paper reports a comprehensive scientific survey and experimental evaluation process. The thorough selection of relevant scientific articles is complemented by an experimental assessment process which has the role to validate the essential claims made by the papers that are included into this survey. Significant numerical results of the experimental process, optimal values of certain technical parameters, and the biochemical particulars of the conducted experiments are reported in Section 2, Section 3 and Section 4. Therefore, this paper may constitute a useful reference concerning the up-to-date academic and industrial developments regarding the available types of biosensors, their technical features, their relevance to academic, research, medical, and general industrial real-world use case scenarios, and the remaining problems that should be addressed by future research and development processes.

The commercial application of electrochemical biosensors in general and to the scope of wearable devices in particular requires consistent fundamental and applied research efforts conducted by both the academia and the interested industry actors. These should concern the optimization of the biosensing techniques and the improvement of the manufacturing materials considering their structural flexibility. Additionally, the set of electronic components and technologies should be consolidated, both functionally and logically. The ultimate goal is to improve the integration of the relevant biosensors into the target wearable devices, while the biological data collection, processing, and storage would conform to efficient, reliable, and secure models.

## References

[1] Clark L.C.J., Lyons C. (1962). Electrode systems for continuous monitoring in cardiovascular surgery. Ann. N. Y. Acad. Sci..

[2] Lambrianou A., Demin S., Hall E.A.H., Renneberg R., Lisdat F. (2007). Biosensing for the 21st Century.

[3] Chain C.Y., Franchin L., Cisneros J.S., Villagra A.P., Labriola C.A., Paccagnella A., Bonaldo S. (2023). Impedimetric Screen-Printed Immunosensor for the Rapid Detection of Chagas Disease. IEEE Sens. J..

[4] Hasan M.R., Ahommed M.S., Daizy M., Bacchu M.S., Ali M.R., Al-Mamun M.R., Aly M.A.S., Khan M.Z.H., Hossain S.I. (2021). Recent development in electrochemical biosensors for cancer biomarkers detection. Biosens. Bioelectron. X.

[5] Bonaldo S., Franchin L., Cretaio E., Pasqualotto E., Scaramuzza M., Paccagnella A. (2024). Electrochemical Biosensor for the Monitoring of Phages of Lactococcus lactis in Milk-Based Samples. IEEE Sens. J..

[6] Labib M., Sargent E.H., Kelley S.O. (2016). Electrochemical methods for the analysis of clinically relevant biomolecules. Chem. Rev..

[7] Wu J., Fu Z.F., Yan F., Ju H.X. (2007). Biomedical and clinical applications of immunoassays and immunosensors for tumor markers. Trends Anal. Chem..

[8] Minteer S.D. (2018). Advances in electroanalytical chemistry. J. Am. Chem. Soc..

[9] Zhang J.J., Xiang Y., Wang M., Basu A., Lu Y. (2016). Dose-dependent response of personal glucose meters to nicotinamide coenzymes: Applications to point-of-care diagnostics of many non-glucose targets in a single step. Angew. Chem. Int. Ed..

[10] Das A., Cui X.K., Chivukula V., Iyer S.S. (2018). Detection of enzymes, viruses, and bacteria using glucose meters. Anal. Chem..

[11] Gong S.H., Li J.J., Pan W., Li N., Tang B. (2021). Duplex-specific nuclease-assisted CRISPRCas12a strategy for microRNA detection using a personal glucose meter. Anal. Chem..

[12] Liu R., Hu Y.S., He Y., Lan T., Zhang J.J. (2021). Translating daily COVID-19 screening into a simple glucose test: A proof of concept study. Chem. Sci..

[13] Cao Y., Mo F., Liu Y., Liu Y., Li G., Yu W., Liu X. (2022). Portable and sensitive detection of non-glucose target by enzyme-encapsulated metal-organic-framework using personal glucose meter. Biosens. Bioelectron..

[14] Lubin A.A., Plaxco K.W. (2010). Folding-based electrochemical biosensors: The case for responsive nucleic acid architectures. Acc. Chem. Res..

[15] Zhang H.Q., Li F., Dever B., Li X.F., Le X.C. (2013). DNA-mediated homogeneous binding assays for nucleic acids and proteins. Chem. Rev..

[16] Liu B.W., Du D., Hua X., Yu X.Y., Lin Y.H. (2014). Paper-based electrochemical biosensors: From test strips to paper-based microfluidics. Electroanalysis.

[17] Sassa F., Biswas G.C., Suzuki H. (2020). Microfabricated electrochemical sensing devices. Lab Chip.

[18] Noviana E., McCord C.P., Clark K.M., Jang I., Henry C.S. (2020). Electrochemical paper-based devices: Sensing approaches and progress toward practical applications. Lab Chip.

[19] Lei J.P., Ju H.X. (2012). Signal amplification using functional nanomaterials for biosensing. Chem. Soc. Rev..

[20] Ju H.X. (2012). Biosensors: Signal amplification for highly sensitive bioanalysis based on biosensors or biochips. J. Biochips Tissue Chips.

[21] Heikenfeld J., Jajack A., Feldman B., Granger S.W., Gaitonde S., Begtrup G., Katchman B.A. (2019). Accessing analytes in biofluids for peripheral biochemical monitoring. Nat. Biotech..

[22] Yu Y., Nyein H.Y.Y., Gao W., Javey A. (2020). Flexible electrochemical bioelectronics: The rise of in situ bioanalysis. Adv. Mater..

[23] Xu C.H., Yang Y.R., Gao W. (2020). Skin-interfaced sensors in digital medicine: From materials to applications. Matter.

[24] Song Y., Min J.H., Gao W. (2019). Wearable and implantable electronics: Moving toward precision therapy. ACS Nano.

[25] Liu Z., Zhao Y., Yin Z. (2024). Low-power soft transistors triggering revolutionary electronics. Innovation.

[26] Zhang T., Liu N., Xu J., Liu Z., Zhou Y., Yang Y., Li S., Huang Y., Jiang S. (2023). Flexible electronics for cardiovascular healthcare monitoring. Innovation.

[27] Hussein H.A., Kandeil A., Gomaa M., Mohamed El Nashar R., El-Sherbiny I.M., Hassan R.Y. (2021). SARS-CoV-2-impedimetric biosensor: Virus-imprinted chips for early and rapid diagnosis. ACS Sens..

[28] Zeng J., Duarte P.A., Ma Y., Savchenko O., Shoute L., Khaniani Y., Babiuk S., Zhuo R., Abdelrasoul G.N., Charlton C. (2022). An impedimetric biosensor for COVID-19 serology test and modification of sensor performance via dielectrophoresis force. Biosen. Bioelectron..

[29] Karyakin A.A., Gitelmacher O.V., Karyakina E.E. (1995). Prussian blue-based first-generation biosensor: A sensitive amperometric electrode for glucose. Anal. Chem..

[30] Ju H.X., Zhang X.J., Wang J. (2011). NanoBiosensing: Principles, Development and Application.

[31] Kim S.K., Lee G.H., Jeon C., Han H.H., Kim S.J., Mok J.W., Joo C.K., Shin S., Sim J.Y., Myung D. (2022). Bimetallic nanocatalysts immobilized in nanoporous hydrogels for long-term robust continuous glucose monitoring of smart contact lens. Adv. Mater..

[32] Huang Y.Z., Han Y.K., Sun J.Y., Zhang Y., Han L. (2022). Dual nanocatalysts co-decorated three-dimensional, laser-induced graphene hybrid nanomaterials integrated with a smartphone portable electrochemical system for point-of-care non-enzymatic glucose diagnosis. Mater. Today Chem..

[33] Wu J., Tang J., Dai Z., Yan F., Ju H., El Murr N. (2006). A disposable electrochemical immunosensor for flow injection immunoassay of carcinoembryonic antigen. Biosens. Bioelectron..

[34] Wu J., Yan F., Tang J.H., Zhai C., Ju H.X. (2007). A disposable multianalyte electrochemical immunosensor array for automated simultaneous determination of tumor markers. Clin. Chem..

[35] Wu J., Yan F., Zhang X., Yan Y., Tang J., Ju H. (2008). Disposable reagentless electrochemical immunosensor array based on a biopolymer/sol-gel membrane for simultaneous measurement of several tumor markers. Clin. Chem..

[36] Fernandez-la-Villa A., Pozo-Ayuso D.F., Castano-Alvarez M. (2019). Microfluidics and electrochemistry: An emerging tandem for next-generation analytical microsystems. Curr. Opin. Electrochem..

[37] Kikkeri K., Wu D., Voldman J. (2022). A sample-to-answer electrochemical biosensor system for biomarker detection. Lab Chip.

[38] Koklu A., Wustoni S., Musteata V.E., Ohayon D., Moser M., McCulloch I., Nunes S.P., Inal S. (2021). Microfluidic integrated organic electrochemical transistor with a nanoporous membrane for amyloid-beta detection. ACS Nano.

[39] Sun S., Luo J., Zhu Y., Kong F., Mao G., Ming T., Xing Y., Liu J., Dai Y., Yan S. (2022). Multifunctional self-driven origami paper-based integrated microfluidic chip to detect CRP and PAB in whole blood. Biosens. Bioelectron..

[40] Feng D., Su J., Xu Y., He G., Wang C., Wang X., Pan T., Ding X., Mi X. (2021). DNA tetrahedron-mediated immune-sandwich assay for rapid and sensitive detection of PSA through a microfluidic electrochemical detection system. Microsyst. Nanoeng..

[41] Lee G., Lee J., Kim J., Choi H.S., Kim J., Lee S., Lee H. (2017). Single microfluidic electrochemical sensor system for simultaneous multi-pulmonary hypertension biomarker analyses. Sci. Rep..

[42] Pursey J.P., Chen Y., Stulz E., Park M.K., Kongsuphol P. (2017). Microfluidic electrochemical multiplex detection of bladder cancer DNA markers. Sens. Actuators B Chem..

[43] Fragoso A., Latta D., Laboria N., von Germar F., Hansen-Hagge T.E., Kemmner W., Gärtner C., Klemm R., Drese K.S., O’Sullivan C.K. (2011). Integrated microfluidic platform for the electrochemical detection of breast cancer markers in patient serum samples. Lab Chip.

[44] Zhao Y.X., Chen F., Li Q., Wang L.H., Fan C.H. (2015). Isothermal amplification of nucleic acids. Chem. Rev..

[45] Simmel F.C., Yurke B., Singh H.R. (2019). Principles and applications of nucleic acid strand displacement reactions. Chem. Rev..

[46] Dai Y., Somoza R.A., Wang L., Welter J.F., Li Y., Caplan A.I., Liu C.C. (2019). Exploring the trans-cleavage activity of CRISPR-Cas12a (cpf1) for the development of a universal electrochemical biosensor. Angew. Chem. Int. Ed..

[47] Zhang D., Yan Y., Que H., Yang T., Cheng X., Ding S., Zhang X., Cheng W. (2020). CRISPR/Cas12a-mediated interfacial cleaving of hairpin DNA reporter for electrochemical nucleic acid sensing. ACS Sens..

[48] Hu J.M., Wang T.Y., Kim J., Shannon C., Easley C.J. (2012). Quantitation of femtomolar protein levels via direct readout with the electrochemical proximity assay. J. Am. Chem. Soc..

[49] Hu J., Yu Y., Brooks J.C., Godwin L.A., Somasundaram S., Torabinejad F., Kim J., Shannon C., Easley C.J. (2014). A reusable electrochemical proximity assay for highly selective, real-time protein quantitation in biological matrices. J. Am. Chem. Soc..

[50] Ren K.W., Wu J., Yan F., Ju H.X. (2014). Ratiometric electrochemical proximity assay for sensitive one-step protein detection. Sci. Rep..

[51] Ren K.W., Wu J., Yan F., Zhang Y., Ju H.X. (2015). Immunoreaction-triggered DNA assembly for one-step sensitive ratiometric electrochemical biosensing of protein biomarker. Biosens. Bioelectron..

[52] Ren K.W., Wu J., Zhang Y., Yan F., Ju H.X. (2014). Proximity hybridization regulated DNA biogate for sensitive electrochemical immunoassay. Anal. Chem..

[53] Ren K.W., Wu J., Ju H.X., Yan F. (2015). Target-driven triple-binder assembly of MNAzyme for amplified electrochemical immunosensing of protein biomarker. Anal. Chem..

[54] Zhu J., Gan H.Y., Wu J., Ju H.X. (2018). Molecular machine powered surface programmatic chain reaction for highly sensitive electrochemical detection of protein. Anal. Chem..

[55] Man Y., Liu J., Wu J., Yin L., Pei H., Wu Q., Xia Q., Ju H. (2020). An anchored monopodial DNA walker triggered by proximity hybridization for amplified amperometric biosensing of nucleic acid and protein. Anal. Chim. Acta.

[56] Karimi-Maleh H., Orooji Y., Karimi F., Alizadeh M., Baghayeri M., Rouhi J., Tajik S., Beitollahi H., Agarwal S., Gupta V.K. (2021). A critical review on the use of potentiometric based biosensors for biomarkers detection. Biosens. Bioelectron..

[57] Ding J.W., Chen Y., Wang X.W., Qin W. (2012). Label-free and substrate-free potentiometric aptasensing using polycation-sensitive membrane electrodes. Anal. Chem..

[58] Nurlely A.M., Heng L.Y., Tan L.L. (2021). Potentiometric enzyme biosensor for rapid determination of formaldehyde based on succinimide-functionalized polyacrylate ion-selective membrane. Measurement.

[59] Özbek O., Berkel C., Isildak Ö., Isildak I. (2022). Potentiometric urea biosensors. Clin. Chim. Acta.

[60] Wu J., Chumbimuni-Torres K.Y., Galik M., Thammakhet C., Haake D.A., Wang J. (2009). Potentiometric detection of DNA hybridization using enzyme-induced metallization and a silver ion selective electrode. Anal. Chem..

[61] Ding J.W., Li B.W., Chen L.X., Qin W. (2016). A three-dimensional origami paper-based device for potentiometric biosensing. Angew. Chem. Int. Ed..

[62] Tang H., Yan F., Lin P., Xu J.B., Chan H.L.W. (2011). Highly sensitive glucose biosensors based on organic electrochemical transistors using platinum gate electrodes modified with enzyme and nanomaterials. Adv. Funct. Mater..

[63] Mak C.H., Liao C., Fu Y., Zhang M., Tang C.Y., Tsang Y.H., Chan H.L., Yan F. (2015). Highly-sensitive epinephrine sensors based on organic electrochemical transistors with carbon nanomaterial modified gate electrodes. J. Mater. Chem. C.

[64] Parlak O., Keene S.T., Marais A., Curto V.F., Salleo A. (2018). Molecularly selective nanoporous membrane-based wearable organic electrochemical device for noninvasive cortisol sensing. Sci. Adv..

[65] Lin P., Luo X.T., Hsing I.M., Yan F. (2011). Organic electrochemical transistors integrated in flexible microfluidic systems and used for label-free DNA sensing. Adv. Mater..

[66] Fu Y., Wang N., Yang A., Law H.K.W., Li L., Yan F. (2017). Highly sensitive detection of protein biomarkers with organic electrochemical transistors. Adv. Mater..

[67] Kim D.J., Lee N.E., Park J.S., Park I.J., Kim J.G., Cho H.J. (2010). Organic electrochemical transistor based immunosensor for prostate specific antigen (PSA) detection using gold nanoparticles for signal amplification. Biosens. Bioelectron..

[68] He R.X., Zhang M., Tan F., Leung P.H., Zhao X.Z., Chan H.L., Yang M., Yan F. (2012). Detection of bacteria with organic electrochemical transistors. J. Mater. Chem..

[69] Lin B.P., Yan F., Yu J.J., Chan H.L.W., Yang M. (2010). The application of organic electrochemical transistors in cell-based biosensors. Adv. Mater..

[70] Chen L., Fu Y., Wang N., Yang A., Li Y., Wu J., Ju H., Yan F. (2018). Organic electrochemical transistors for the detection of cell surface glycans. ACS Appl. Mater. Interfaces.

[71] Chen L.Z., Wang N.X., Wu J., Yan F., Ju H.X. (2020). Organic electrochemical transistor for sensing of sialic acid in serum samples. Anal. Chim. Acta.

[72] Chen L.Z., Wu J., Yan F., Ju H.X. (2021). A facile strategy for quantitative sensing of glycans on cell surface using organic electrochemical transistors. Biosens. Bioelectron..

[73] Chen L.Z., Wu J., Yan F., Ju H.X. (2021). Monose-modified organic electrochemical transistors for cell surface glycan analysis via competitive recognition to enzyme-labeled lectin. Mikrochim. Acta.

[74] Wang N., Yang A., Fu Y., Li Y., Yan F. (2019). Functionalized organic thin film transistors for biosensing. Acc. Chem. Res..

[75] Yang A., Li Y., Yang C., Fu Y., Wang N., Li L., Yan F. (2018). Fabric organic electrochemical transistors for biosensors. Adv. Mater..

[76] Zang Y., Lei J.P., Ju H.X. (2017). Principles and applications of photoelectrochemical sensing strategies based on biofunctionalized nanostructures. Biosens. Bioelectron..

[77] Ruan Y.F., Chen F.Z., Xu Y.T., Zhang T.Y., Yu S.Y., Zhao W.W., Jiang D., Chen H.Y., Xu J.J. (2021). An integrated photoelectrochemical nanotool for intracellular drug delivery and evaluation of treatment effect. Angew. Chem. Int. Ed..

[78] Hu F.X., Miao J.W., Guo C.X., Yang H.B., Liu B. (2021). Real-time photoelectrochemical quantification of hydrogen peroxide produced by living cells. Chem. Eng. J..

[79] Ye X., Wang X., Kong Y., Dai M., Han D., Liu Z. (2021). FRET modulated signaling: A versatile strategy to construct photoelectrochemical microsensors for in vivo analysis. Angew. Chem. Int. Ed..

[80] Qi H.L., Zhang C.X. (2020). Electrogenerated chemiluminescence biosensing. Anal. Chem..

[81] Chen Y.H., Ding Z.F. (2022). Highly sensitive analysis strategies of microRNAs based on electrochemiluminescence. Curr. Opin. Electrochem..

[82] Miao W.J. (2008). Electrogenerated chemiluminescence and its biorelated applications. Chem. Rev..

[83] Delaney J.L., Hogan C.F., Tian J., Shen W. (2011). Electrogenerated chemiluminescence detection in paper-based microfluidic sensors. Anal. Chem..

[84] Kerr E., Farr R., Doeven E.H., Nai Y.H., Alexander R., Guijt R.M., Prieto-Simon B., Francis P.S., Dearnley M., Hayne D.J. (2021). Amplification-free electrochemiluminescence molecular beacon-based microRNA sensing using a mobile phone for detection. Sens. Actuators B Chem..

[85] Deiss F., LaFratta C.N., Symer M., Blicharz T.M., Sojic N., Walt D.R. (2009). Multiplexed sandwich immunoassays using electrochemiluminescence imaging resolved at the single bead level. J. Am. Chem. Soc..

[86] Wang N.N., Chen L.Z., Chen W.W., Ju H.X. (2021). Potential-and color-resolved electrochemiluminescence of polymer dots for array imaging of multiplex microRNAs. Anal. Chem..

[87] Ma C., Cao Y., Gou X.D., Zhu J.J. (2020). Recent progress in electrochemiluminescence sensing and imaging. Anal. Chem..

[88] Wang N., Gao H., Li Y., Li G., Chen W., Jin Z., Lei J., Wei Q., Ju H. (2021). Dual intramolecular electron transfer for in situ coreactant-embedded electrochemiluminescence microimaging of membrane protein. Angew. Chem. Int. Ed..

[89] Wang N., Ao H., Xiao W., Chen W., Li G., Wu J., Ju H. (2022). Confined electrochemiluminescence imaging microarray for high-throughput biosensing of single cell-released dopamine. Biosens. Bioelectron..

[90] Teymourian H., Barfidokht A., Wang J. (2020). Electrochemical glucose sensors in diabetes management: An updated review (2010–2020). Chem. Soc. Rev..

[91] Nagata R., Yokoyama K., Clark S.A., Karube I. (1995). A glucose sensor fabricated by the screen printing technique. Biosens. Bioelectron..

[92] Wang J. (2008). Electrochemical glucose biosensors. Chem. Rev..

[93] Tonyushkina K., Nichols J.H. (2009). Glucose meters: A review of technical challenges to obtaining accurate results. J. Diabetes Sci. Technol..

[94] Karon B.S., Boyd J.C., Klee G.G. (2010). Glucose meter performance criteria for tight glycemic control estimated by simulation modeling. Clin. Chem..

[95] Xiang Y., Lu Y. (2011). Using personal glucose meters and functional DNA sensors to quantify a variety of analytical targets. Nat. Chem..

[96] Zhang J., Lu Y. (2018). Biocomputing for portable, resettable, and quantitative point-of-care diagnostics: Making the glucose meter a logic-gate responsive device for measuring many clinically relevant targets. Angew. Chem. Int. Ed..

[97] Amalfitano E., Karlikow M., Norouzi M., Jaenes K., Cicek S., Masum F., Sadat Mousavi P., Guo Y., Tang L., Sydor A. (2021). A glucose meter interface for point-of-care gene circuit-based diagnostics. Nat. Commun..

[98] Zhang X., Dhawane A.N., Sweeney J., He Y., Vasireddi M. (2015). Electrochemical assay to detect influenza viruses and measure drug susceptibility. Angew. Chem. Int. Ed..

[99] Ahn J.K., Kim H.Y., Park K.S., Park H.G. (2018). A personal glucose meter for label-free and washing-free biomolecular detection. Anal. Chem..

[100] Singh N.K., Ray P., Carlin A.F., Magallanes C., Morgan S.C., Laurent L.C., Aronoff-Spencer E.S., Hall D.A. (2021). Hitting the diagnostic sweet spot: Point-of-care SARS-CoV-2 salivary antigen testing with an off-the-shelf glucometer. Biosens. Bioelectron..

[101] Rackus D.G., Shamsi M.H., Wheeler A.R. (2015). Electrochemistry, biosensors and microfluidics: A convergence of fields. Chem. Soc. Rev..

[102] Zhao H., Zhang Y., Chen Y., Ho N.R., Sundah N.R., Natalia A., Liu Y., Miow Q.H., Wang Y., Tambyah P.A. (2021). Accessible detection of SARS-CoV-2 through molecular nanostructures and automated microfluidics. Biosens. Bioelectron..

[103] Gong M.M., Sinton D. (2017). Turning the page: Advancing paper-based microfluidics for broad diagnostic application. Chem. Rev..

[104] Liu H., Xiang Y., Lu Y., Crooks R.M. (2012). Aptamer-based origami paper analytical device for electrochemical detection of adenosine. Angew. Chem. Int. Ed..

[105] Su J., Chen S., Dou Y., Zhao Z., Jia X., Ding X., Song S. (2022). Smartphone-based electrochemical biosensors for directly detecting serum-derived exosomes and monitoring their secretion. Anal. Chem..

[106] Guo J.H. (2018). Smartphone-powered electrochemical biosensing dongle for emerging medical IoTs application. IEEE T. Ind. Inform..

[107] Ainla A., Mousavi M.P., Tsaloglou M.N., Redston J., Bell J.G., Fernandez-Abedul M.T., Whitesides G.M. (2018). Open-source potentiostat for wireless electrochemical detection with smartphones. Anal. Chem..

[108] Kim J., Campbell A.S., de Avila B.E., Wang J. (2019). Wearable biosensors for healthcare monitoring. Nat. Biotechnol..

[109] Moyer J., Wilson D., Finkelshtein I., Wong B., Potts R. (2012). Correlation between sweat glucose and blood glucose in subjects with diabetes. Diabetes Technol. Ther..

[110] Baker L.B. (2019). Physiology of sweat gland function: The roles of sweating and sweat composition in human health. Temperature.

[111] Lu Y., Jiang K., Chen D., Shen G.Z. (2019). Wearable sweat monitoring system with integrated micro-supercapacitors. Nano Energy.

[112] Bocu R., Costache C. (2018). A homomorphic encryption-based system for securely managing personal health metrics data. IBM J. Res. Dev..

[113] Quinton P.M. (1983). Sweating and its disorders. Annu. Rev. Med..

[114] Choi J., Kang D., Han S., Kim S.B., Rogers J.A. (2017). Thin, soft, skin-mounted microfluidic networks with capillary bursting valves for chrono-sampling of sweat. Adv. Healthc. Mater..

[115] Manjakkal L., Yin L., Nathan A., Wang J., Dahiya R. (2021). Energy autonomous sweat-based wearable systems. Adv. Mater..

[116] Lou Z., Wang L.L., Shen G.Z. (2018). Recent advances in smart wearable sensing systems. Adv. Mater. Technol..

[117] Fu K., Zhou J., Wu H.U., Su Z.Q. (2021). Fibrous self-powered sensor with high stretchability for physiological information monitoring. Nano Energy.

[118] Jia W., Valdes-Ramirez G., Bandodkar A.J., Windmiller J.R., Wang J. (2013). Epidermal biofuel cells: Energy harvesting from human perspiration. Angew. Chem. Int. Ed..

[119] Falk M., Andoralov V., Silow M., Toscano M.D., Shleev S. (2013). Miniature biofuel cell as a potential power source for glucose-sensing contact lenses. Anal. Chem..

[120] Chen Z., Xi J., Huang W., Yuen M.M.F. (2017). Stretchable conductive elastomer for wireless wearable communication applications. Sci. Rep..

[121] Lukocius R., Vaitkunas M., Virbalis J.A., Dosinas A., Vegys A. (2014). Physiological parameters monitoring system for occupational safety. Elektron. Elektrotech..

[122] Bujes-Garrido J., Arcos-Martínez M.J. (2017). Development of a wearable electrochemical sensor for voltammetric determination of chloride ions. Sens. Actuators B Chem..

[123] Vaddiraju S., Burgess D.J., Tomazos I., Jain F.C., Papadimitrakopoulos F. (2010). Technologies for continuous glucose monitoring: Current problems and future promises. J. Diabetes Sci. Technol..

[124] Bobrowski T., Schuhmann W. (2018). Long-term implantable glucose biosensors. Curr. Opin. Electrochem..

[125] Dalrymple A.N. (2021). Implanted devices: The importance of both electrochemical performance and biological acceptance. Neural Regen. Res..

[126] Kotanen C.N., Moussy F.G., Carrara S., Guiseppi-Elie A. (2012). Implantable enzyme amperometric biosensors. Biosens. Bioelectron..

[127] Wagner J., Tennen H., Wolpert H. (2012). Continuous glucose monitoring: A review for behavioral researchers. Psychosom. Med..

[128] McGarraugh G. (2009). The chemistry of commercial continuous glucose monitors. Diabetes Technol. Ther..

[129] Lee H., Hong Y.J., Baik S., Hyeon T., Kim D.H. (2018). Enzyme-based glucose sensor: From invasive to wearable device. Adv. Healthc. Mater..

[130] Tavakolian-Ardakani Z., Hosu O., Cristea C., Mazloum-Ardakani M., Marrazza G. (2019). Latest trends in electrochemical sensors for neurotransmitters: A review. Sensors.

[131] Seaton B.T., Heien M.L. (2021). Biocompatible reference electrodes to enhance chronic electrochemical signal fidelity in vivo. Anal. Bioanal. Chem..

[132] Wickramasinghe Y., Yang Y., Spencer S.A. (2004). Current problems and potential techniques in in vivo glucose monitoring. J. Fluoresc..

[133] Nichols S.P., Koh A., Storm W.L., Shin J.H., Schoenfisch M.H. (2013). Biocompatible materials for continuous glucose monitoring devices. Chem. Rev..

[134] Malone-Povolny M.J., Merricks E.P., Wimsey L.E., Nichols T.C., Schoenfisch M.H. (2019). Long-term accurate continuous glucose biosensors via extended nitric oxide release. ACS Sens..

[135] Hetrick E.M., Schoenfisch M.H. (2006). Reducing implant-related infections: Active release strategies. Chem. Soc. Rev..

[136] Ben Amar A., Kouki A.B., Cao H. (2015). Power approaches for implantable medical devices. Sensors.

[137] Rebelo R., Barbosa A.I., Correlo V.M., Reis R.L. (2021). An outlook on implantable biosensors for personalized medicine. Engineering.

[138] Yang Y., Wei X.J., Liu J. (2007). Suitability of a thermoelectric power generator for implantable medical electronic devices. J. Phys. D Appl. Phys..

[139] Simons P., Schenk S.A., Gysel M.A., Olbrich L.F., Rupp J.L.M. (2022). A ceramic-electrolyte glucose fuel cell for implantable electronics. Adv. Mater..

[140] Scholten K., Meng E. (2018). A review of implantable biosensors for closed-loop glucose control and other drug delivery applications. Int. J. Pharm..

[141] Green R.A., Lovell N.H., Wallace G.G., Poole-Warren L.A. (2008). Conducting polymers for neural interfaces: Challenges in developing an effective long-term implant. Biomaterials.

[142] Kutner N., Kunduru K.R., Rizik L., Farah S. (2021). Recent advances for improving functionality, biocompatibility, and longevity of implantable medical devices and deliverable drug delivery systems. Adv. Funct. Mater..

[143] Hsieh K., Ferguson B.S., Eisenstein M., Plaxco K.W., Soh H.T. (2015). Integrated electrochemical microsystems for genetic detection of pathogens at the point of care. Acc. Chem. Res..

[144] Zhang J., Lan T., Lu Y. (2020). Translating in vitro diagnostics from centralized laboratories to point-of-care locations using commercially-available handheld meters. Trends Anal. Chem..

[145] Loncaric C., Tang Y.T., Ho C., Parameswaran M.A., Yu H.Z. (2012). A USB-based electrochemical biosensor prototype for point-of-care diagnosis. Sens. Actuators B Chem..

[146] Tu J.B., Torrente-Rodriguez R.M., Wang M.Q., Gao W. (2020). The era of digital health: A review of portable and wearable affinity biosensors. Adv. Funct. Mater..

[147] Gordon W.J., Stern A.D. (2019). Challenges and opportunities in software-driven medical devices. Nat. Biomed. Eng..

[148] Liu H., Zhao C. (2020). Wearable electrochemical sensors for noninvasive monitoring of health-a perspective. Curr. Opin. Electrochem..

[149] Dixon A.M., Allstot E.G., Gangopadhyay D., Allstot D.J. (2012). Compressed sensing system considerations for ECG and EMG wireless biosensors. IEEE Trans. Biomed. Circuits Syst..

[150] Martín A., Kim J., Kurniawan J.F., Sempionatto J.R., Moreto J.R., Tang G., Campbell A.S., Shin A., Lee M.Y., Liu X. (2017). Epidermal microfluidic electrochemical detection system: Enhanced sweat sampling and metabolite detection. ACS Sens..

[151] Tang H., Lin P., Chan H.L.W., Yan F. (2011). Highly sensitive dopamine biosensors based on organic electrochemical transistors. Biosens. Bioelectron..

[152] Rose D.P., Ratterman M.E., Griffin D.K., Hou L., Kelley-Loughnane N., Naik R.R., Hagen J.A., Papautsky I., Heikenfeld J.C. (2015). Adhesive RFID sensor patch for monitoring of sweat electrolytes. IEEE Trans. Biomed. Eng..

[153] Keum D.H., Kim S.K., Koo J., Lee G.H., Jeon C., Mok J.W., Mun B.H., Lee K.J., Kamrani E., Joo C.K. (2020). Wireless smart contact lens for diabetic diagnosis and therapy. Sci. Adv..

[154] Lee K., Ni X., Lee J.Y., Arafa H., Pe D.J., Xu S., Avila R., Irie M., Lee J.H., Easterlin R.L. (2020). Mechano-acoustic sensing of physiological processes and body motions via a soft wireless device placed at the suprasternal notch. Nat. Biomed. Eng..

[155] Li P., Lee G.H., Kim S.Y., Kwon S.Y., Kim H.R., Park S. (2021). From diagnosis to treatment: Recent advances in patient-friendly biosensors and implantable devices. ACS Nano.

[156] Li C.M., Dong H., Cao X., Luong J.H., Zhang X. (2007). Implantable electrochemical sensors for biomedical and clinical applications: Progress, problems, and future possibilities. Curr. Med. Chem..

[157] Xu J., Cheng C., Li X., Lu Y., Hu S., Liu G., Zhu L., Wang N., Wang L., Cheng P. (2021). Implantable platinum nanotree microelectrode with a battery-free electrochemical patch for peritoneal carcinomatosis monitoring. Biosens. Bioelectron..

[158] Singh P., Pandey S.K., Singh J., Srivastava S., Sachan S., Singh S.K. (2016). Biomedical perspective of electrochemical nanobiosensor. Nanomicro Lett..

[159] Gross T.M., Bode B.W., Einhorn D., Kayne D.M., Reed J.H., White N.H., Mastrototaro J.J. (2000). Performance evaluation of the MiniMed continuous glucose monitoring system during patient home use. Diabetes Technol. Ther..

[160] Clark J.J., Sandberg S.G., Wanat M.J., Gan J.O., Horne E.A., Hart A.S., Akers C.A., Parker J.G., Willuhn I., Martinez V. (2010). Chronic microsensors for longitudinal, subsecond dopamine detection in behaving animals. Nat. Methods.

[161] Azzouz A., Goud K.Y., Raza N., Ballesteros E., Lee S.E., Hong J., Deep A., Kim K.H. (2019). Nanomaterial-based electrochemical sensors for the detection of neurochemicals in biological matrices. Trends Anal. Chem..

[162] Li J., Liu Y., Yuan L., Zhang B., Bishop E.S., Wang K., Tang J., Zheng Y.Q., Xu W., Niu S. (2022). A tissue-like neurotransmitter sensor for the brain and gut. Nature.

[163] Zheng Q., Shi B., Fan F., Wang X., Yan L., Yuan W., Wang S., Liu H., Li Z., Wang Z.L. (2014). In vivo powering of pacemaker by breathing-driven implanted triboelectric nanogenerator. Adv. Mater..

[164] Hwang G.T., Park H., Lee J.H., Oh S., Park K.I., Byun M., Ahn G., Jeong C.K., No K., Kwon H. (2014). Self-powered cardiac pacemaker enabled by flexible single crystalline PMN-PT piezoelectric energy harvester. Adv. Mater..

